# Supplementary material for: Preanalytical Pitfalls in Untargeted Plasma Nuclear Magnetic Resonance Metabolomics of Endocrine Hypertension
Source: Metabolites. 2022 Jul 24;12(8):679. doi: 10.3390/metabo12080679 (PMC9394285; doi:10.3390/metabo12080679)
Supplement: Supplementary file 1 [file metabolites-12-00679-s001.zip › metabolites-1771207-supplementary.pdf]

metabolites

MDPI

Article

Preanalytical Pitfalls in Untargeted Plasma Nuclear Magnetic Resonance Metabolomics of Endocrine Hypertension

Nikolaos G. Bliziotis [1,*], Leo A. J. Kluijtmans [1,*], Gerjen H. Tinnevelt [2], Parminder Reel [3], Smarti Reel [3], Katharina Langton [4], Mercedes Robledo [5], Christina Pamporaki [4], Alessio Pecori [6], Josie Van Kralingen [7], Martina Tetti [6], Udo F. H. Engelke [1], Zoran Erlic [8], Jasper Engel [9], Timo Deutschbein [10,11], Svenja Nölting [12], Aleksander Prejbisz [13], Susan Richter [14], Jerzy Adamski [15,16,17,18], Andrzej Januszewicz [13], Filippo Ceccato [19], Carla Scaroni [19], Michael C. Dennedy [20], Tracy A. Williams [6], Livia Lenzini [21], Anne-Paule Gimenez-Roqueplo [22,23], Eleanor Davies [7], Martin Fassnacht [10,24,25], Hanna Remde [10], Graeme Eisenhofer [4,14], Felix Beuschlein [8,12], Matthias Kroiss [10,12,24,25], Emily Jefferson [3,26], Maria-Christina Zennaro [22,23], Ron A. Wevers [1,*], Jeroen J. Jansen [2], Jaap Deinum [27] and Henri J. L. M. Timmers [27,*]

1 Department of Laboratory Medicine, Translational Metabolic Laboratory, Radboud University Medical Center, 6525 GA Nijmegen, The Netherlands; udo.engelke@radboudumc.nl
2 Department of Analytical Chemistry, Institute for Molecules and Materials, Radboud University, 6500 HB Nijmegen, The Netherlands; g.tinnevelt@science.ru.nl (G.H.T.); jeroen.jansen@ru.nl (J.J.J.)
3 Division of Population Health and Genomics, School of Medicine, University of Dundee, Dundee DD2 4BF, UK; p.s.reel@dundee.ac.uk (P.R.); s.reel@dundee.ac.uk (S.R.); e.r.jefferson@dundee.ac.uk (E.J.)
4 Department of Medicine III, University Hospital Carl Gustav Carus, Technische Universität Dresden, 01307 Dresden, Germany; katharina.langton@uniklinikum-dresden.de (K.L.); christina.pamporaki@uniklinikum-dresden.de (C.P.); graeme.eisenhofer@uniklinikum-dresden.de (G.E.)
5 Hereditary Endocrine Cancer Group, Spanish National Cancer Research Centre (CNIO), and Centro de Investigación Biomédica en Red de Enfermedades Raras (CIBERER), 28029 Madrid, Spain; mrobledo@cnio.es
6 Division of Internal Medicine and Hypertension Unit, Department of Medical Sciences, University of Torino, 10124 Torino, Italy; alessio.pecori@edu.unito.it (A.P.); tetti.martina@gmail.com (M.T.); tracyannwilliams48@gmail.com (T.A.W.)
7 British Heart Foundation Glasgow Cardiovascular Research Centre (BHF GCRC), Institute of Cardiovascular & Medical Sciences (ICAMS), University of Glasgow, Glasgow G12 8TA, UK; josievankralingen@googlemail.com (J.V.K.); eleanor.davies@glasgow.ac.uk (E.D.)
8 Department of Endocrinology, Diabetology and Clinical Nutrition, University Hospital Zurich (USZ) and University of Zurich (UZH), 8091 Zurich, Switzerland; zoran.erlic@usz.ch (Z.E.); felix.beuschlein@usz.ch (F.B.)
9 Biometris, Wageningen University & Research, 6708 PB Wageningen, The Netherlands; jasper.engel@wur.nl
10 Department of Internal Medicine I, Division of Endocrinology and Diabetes, University Hospital, University of Würzburg, 97080 Wurzburg, Germany; deutschbein_t@ukw.de (T.D.); fassnacht_m@ukw.de (M.F.); remde_h@ukw.de (H.R.); kroiss_m@ukw.de (M.K.)
11 Medicover Oldenburg MVZ, 26122 Oldenburg, Germany
12 Department of Medicine IV, University Hospital, LMU Munich, 80336 Munich, Germany; svenja.noelting@med.uni-muenchen.de
13 Department of Hypertension, Institute of Cardiology, 04-628 Warsaw, Poland; prejbisz@vp.pl (A.P.); ajanu@op.pl (A.J.)
14 Institute of Clinical Chemistry and Laboratory Medicine, University Hospital Carl Gustav Carus at the Technische Universität Dresden, 01307 Dresden, Germany; susan.richter@uniklinikum-dresden.de
15 Research Unit Molecular Endocrinology and Metabolism, Genome Analysis Center, Helmholtz Center München, German Research Center for Environmental Health, D-85764 Neuherberg, Germany; adamski@helmholtz-muenchen.de
16 Institute of Biochemistry, Faculty of Medicine, University of Ljubljana, 1000 Ljubljana, Slovenia
17 Institute of Experimental Genetics, Technical University München, 85350 Freising—Weihenstephan, Germany
18 Department of Biochemistry, Yong Loo Lin School of Medicine, National University of Singapore, Singapore 119077, Singapore
19 Endocrinology Unit, Department of Medicine DIMED, University-Hospital of Padova, 35128 Padova, Italy; ceccato.filippo@gmail.com (F.C.); carla.scaroni@unipd.it (C.S.)
20 The Discipline of Pharmacology and Therapeutics, School of Medicine, National University of Ireland, H91 CF50 Galway, Ireland; conall.dennedy@mailn.hse.ie

Citation: Bliziotis, N.G.; Kluijtmans, L.A.J.; Tinnevelt, G.H.; Reel, P.; Reel, S.; Langton, K.; Robledo, M.; Pamporaki, C.; Pecori, A.; Van Kralingen, J.; et al. Preanalytical Pitfalls in Untargeted Plasma Nuclear Magnetic Resonance Metabolomics of Endocrine Hypertension. *Metabolites* **2022**, *12*, 679. https://doi.org/10.3390/metabo12080679

Academic Editor: Martin Grootveld

Received: 30 May 2022
Accepted: 11 July 2022
Published: 24 July 2022

Publisher's Note: MDPI stays neutral with regard to jurisdictional claims in published maps and institutional affiliations.

Copyright: © 2022 by the authors. Licensee MDPI, Basel, Switzerland. This article is an open access article distributed under the terms and conditions of the Creative Commons Attribution (CC BY) license (https://creativecommons.org/licenses/by/4.0/).

[21] Department of Medicine-DIMED, Emergency and Hypertension Unit, University of Padova, University Hospital, 35126 Padova, Italy; livia.lenzini@gmail.com
[22] INSERM, PARCC, Université de Paris, 75015 Paris, France; anne-paule.gimenez-roqueplo@aphp.fr (A.-P.G.-R.); maria-christina.zennaro@inserm.fr (M.-C.Z.)
[23] Service de Genétique, Assistance Publique-Hôpitaux de Paris, Hôpital Européen Georges Pompidou, 75015 Paris, France
[24] Core Unit Clinical Mass Spectrometry, University Hospital Würzburg, 97080 Würzburg, Germany
[25] Comprehensive Cancer Center Mainfranken, Würzburg University, 97070 Würzburg, Germany
[26] Institute of Health & Wellbeing, Glasgow University, G12 8RZ Glasgow, UK
[27] Department of Internal Medicine, Radboud University Medical Center, 6525 GA Nijmegen, The Netherlands; jaap.deinum@radboudumc.nl (J.D.)
* Correspondence: nick.bliziotis@radboudumc.nl (N.G.B.); leo.kluijtmans@radboudumc.nl (L.A.J.K.); ron.wevers@radboudumc.nl (R.A.W.); henri.timmers@radboudumc.nl (H.J.L.M.T.)

Supplementary Material

Results

PCA of the metabolomics data for all patients, along with HV (n=76) and QC (n=108) samples is shown in Figure S1, with each sample analyzed having a unique metabolic signature that results in a single point in the plot. In total, there was 1 patient with Adrenocortical Carcinoma (ACC), 33 patients with Cushing's Syndrome (CS), 104 patients with Primary Aldosteronism (PA), 106 patients with Primary Hypertension (PHT) and 94 patients with Pheochromocytoma or Paraganglioma (PPGL). According to the plot, in Figure S1, which explains 48% of the dataset's variation, QC samples cluster together closer than samples from any of the other groups, and indicate the amount of analytical variation in the dataset. The coefficients of variation of the final set of 86 peaks in QC samples (which describe technical variation) were multiplied by two to be comparable to those of HVs, PHT, and EHT samples (which describe technical + biological variation) and were found to be lower in QCs than in any of the other groups of samples, with a paired t-test yielding significant results for the comparison of PHT peak CVs (p=1.343e-05), as well as EHT (p=0.004) and the combination of PHT+EHT (p=0.003), but not for HVs (p=0.121). The median coefficient of variation in QC samples was found to be 0.098 in the final dataset, with a median absolute deviation of 0.076. HV samples could be separated based on the batch in which they were analyzed, and QC samples based on their run order within batches. The influence of both these

analytical factors, as well as that of biological factors patient age and sex were not found to be strong influences on data distribution (Figure S2), in contrast to sample center of origin and sample age (Figure 1).

Table S1: Detectable metabolites, based on their corresponding peaks, PubMed CID, level of identification rigor (according to MSI guidelines(Sumner et al., 2007)) and metabolites that also correspond to at least one peak. L2* identification rigor resulted from visual inspection along with 2D NMR and experiments on filtered plasma at pH 2.5.

Metabolite	Peaks (ppm)	CID	Level of Identification rigor	Overlaps with
Acetylcarnitine	3.177	7045767	1	
Alanine	1.457, 1.472	5950	1	
Creatine	3.021, 3.917	586	1	Lysine
Creatinine	3.028, 4.041	588	1	Ornithine
Dimethyl sulfone	3.137	6213	2	
Dimethylamine	2.695	674	2	
Dimethylglycine	2.91	673	2	
Formate	8.441	284	2	
Glucose	5.22, 5.227	5793	2*	
Glutamate	2.047, 2.06, 2.075, 2.095, 2.108, 2.122, 2.145, 2.325, 2.356	33032	2*	Proline, Methionine,

				Glutamine, Pyruvate
Glutamine	2.095, 2.108, 2.122, 2.145, 2.418, 2.428, 2.433, 2.46	5961	2*	Glutamate, Methionine, Proline
Glycerol	3.555, 3.567	753	2*	Threonine, Glycine
Glycine	3.548	750	1	Glycerol
Histidine	3.126, 3.963, 3.973, 3.985	6274	1	Serine, Phenylalanine
Isoleucine	0.907, 0.922, 0.933, 0.987, 1.001	6306	2*	Leucine
Lactate	1.307, 1.321, 4.08, 4.094, 4.108, 4.121	612	2*	Proline, 3-hydroxybutyrate
Leucine	0.933, 0.945, 0.957, 1.71	6106	2*	Isoleucine, Lysine, Ornithine
Lysine	1.71, 1.873, 1.894, 2.997, 3.013, 3.021	5962	1	Creatine, Isoleucine, Ornithine
Methanol	3.346	887	1	Proline
Methionine	2.122	6137	1	Glutamate, Glutamine

Ornithine	1.71, 1.873, 1.894, 3.028, 3.041, 3.057	6262	2*	Creatinine, Lysine, Leucine
Phenylalanine	3.126, 3.963, 3.973, 3.985	6140	1	Histidine, Serine
Proline	1.968, 1.982, 1.996, 2.01, 2.047, 2.06, 2.075, 2.095, 2.325, 3.298, 3.312, 3.321, 3.335, 3.346	145742	2*	Glutamate, Lactate, Methanol
Pyruvate	2.356	1060	1	Glutamate
Serine	3.939, 3.951, 3.963, 3.973, 3.985	5951	1	Histidine, Phenylalanine, Tyrosine
Threonine	3.567, 3.576, 4.227, 4.24, 4.252	6288	1	Glycerol
Tyrosine	3.939, 6.874, 6.892, 7.168, 7.185	6057	1	Serine
Valine	0.967, 0.981, 1.019, 1.033, 2.239, 2.248, 2.253, 3.594, 3.603	6287	2*	
Unknown metabolites	3.162, 3.262, 3.284, 3.612, 3.67		4	

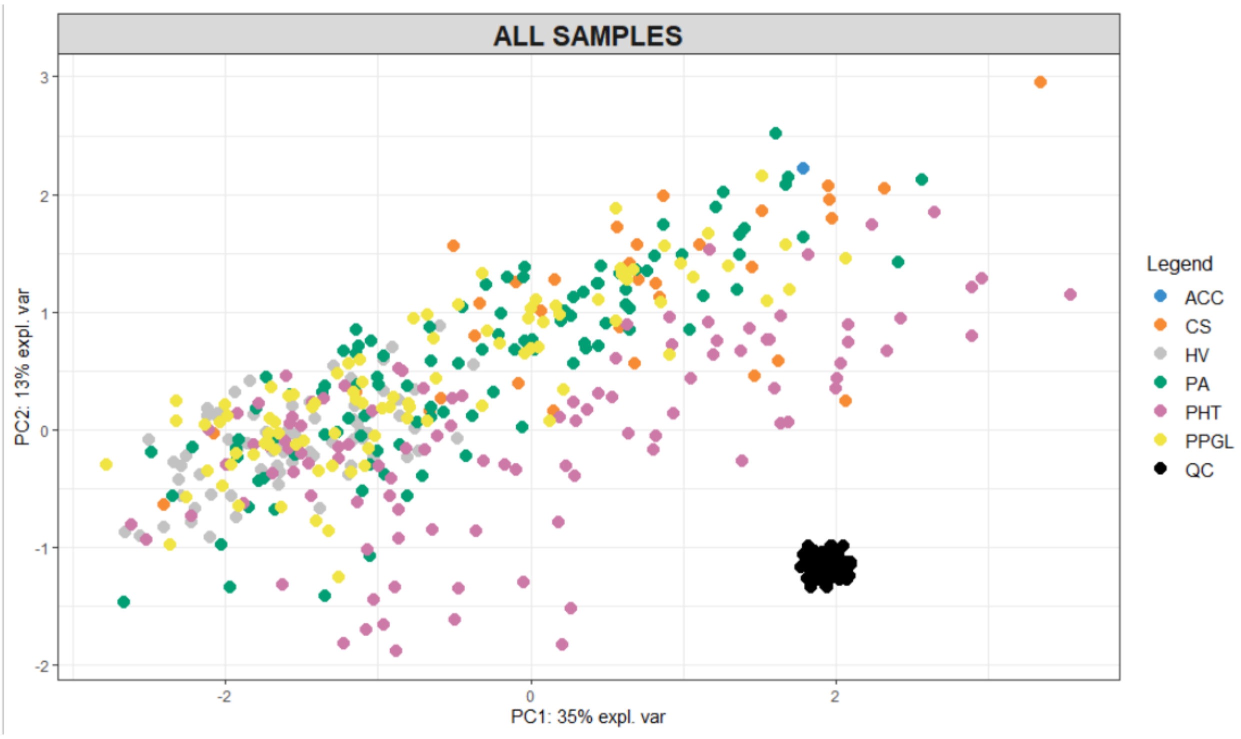

Figure S1: PCA score plot of the complete dataset. This plot includes Healthy Volunteers (HV), Quality Controls (QC), as well as samples collected from all disease groups (PA, PHT, PPGL and CS). One patient was found to have adrenocortical carcinoma (ACC) and was left out of subsequent analyses. Principal components 1 and 2 were used for the plot. QC samples were technical replicates and were aliquoted from pooled plasma collected from patients not included in the present study. The spread of QC samples is indicative of technical variation associated to the data, which is significantly lower than biological.

Table S2: Summary of accuracies from analyses done via the Initial Approach, without correcting for confounders.

Scenario	Analysis	Balanced Accuracy	Sensitivity**	Specificity***	
EHT-PHT	sPLSDA	79[78-79]	87[87-87]	70[70-71]	
	glmnet	77[77-78]	95[95-96]	59[59-60]	

	Including patient age, sex, sPLSDA	79[79-80]	88[88-88]	71[70-71]	
	Including patient age, sex, glmnet	76[76-77]	96[96-96]	57[56-57]	
PA-PHT	sPLSDA	83[83-84]	90[89-90]	77[77-78]	
	glmnet	83[82-83]	86[86-87]	79[79-80]	
	Including patient age, sex, sPLSDA	83[83-84]	90[90-91]	76[76-77]	
	Including patient age, sex, glmnet	84[83-84]	88[88-89]	79[79-79]	
PPGL-PHT	sPLSDA	79[78-79]	88[87-88]	70[69-70]	
	glmnet	78[78-79]	77[76-78]	79[78-80]	
	Including patient age, sex, sPLSDA	78[77-78]	84[83-84]	72[71-72]	

	Including patient age, sex, glmnet	77[77-78]	77[76-78]	78[77-79]		
CS-PHT	sPLSDA	85[84-85]	71[71-72]	98[98-99]$		
	glmnet	84[83-84]	69[68-70]	98[98-99]$		
	Including patient age, sex, sPLSDA	85[84-85]	73[72-74]	97[96-97]		
	Including patient age, sex, glmnet	83[82-83]	67[66-68]	98[98-98]$		
		Balanced Accuracy	CS TP Rate	PA TP* Rate	PHT TP* Rate	PPGL TP* Rate
ALL-ALL	sPLSDA	65[64-65]	73[72-74]	65[64-65]	72[72-72]	50[49-51]
	glmnet	63[62-63]	49[48-50]	69[68-70]	73[73-74]	60[59-61]
	Including patient age, sex, sPLSDA	66[66-67]	76[75-77]	64[63-65]	69[68-69]	57[56-58]
	Including patient age, sex, glmnet	63[62-63]	48[47-49]	70[69-71]	72[72-73]	61[60-62]

*TP stands for True Positive

**Sensitivity is the TP rate of the disease group (EHT, PA, PPGL or CS)

***Specificity is the TP rate of the control group (PHT).

All metrics are given as means, with the 95% confidence interval (in brackets). The marked populations of CS-PHT model estimates ($) were not normal (values close to 100).

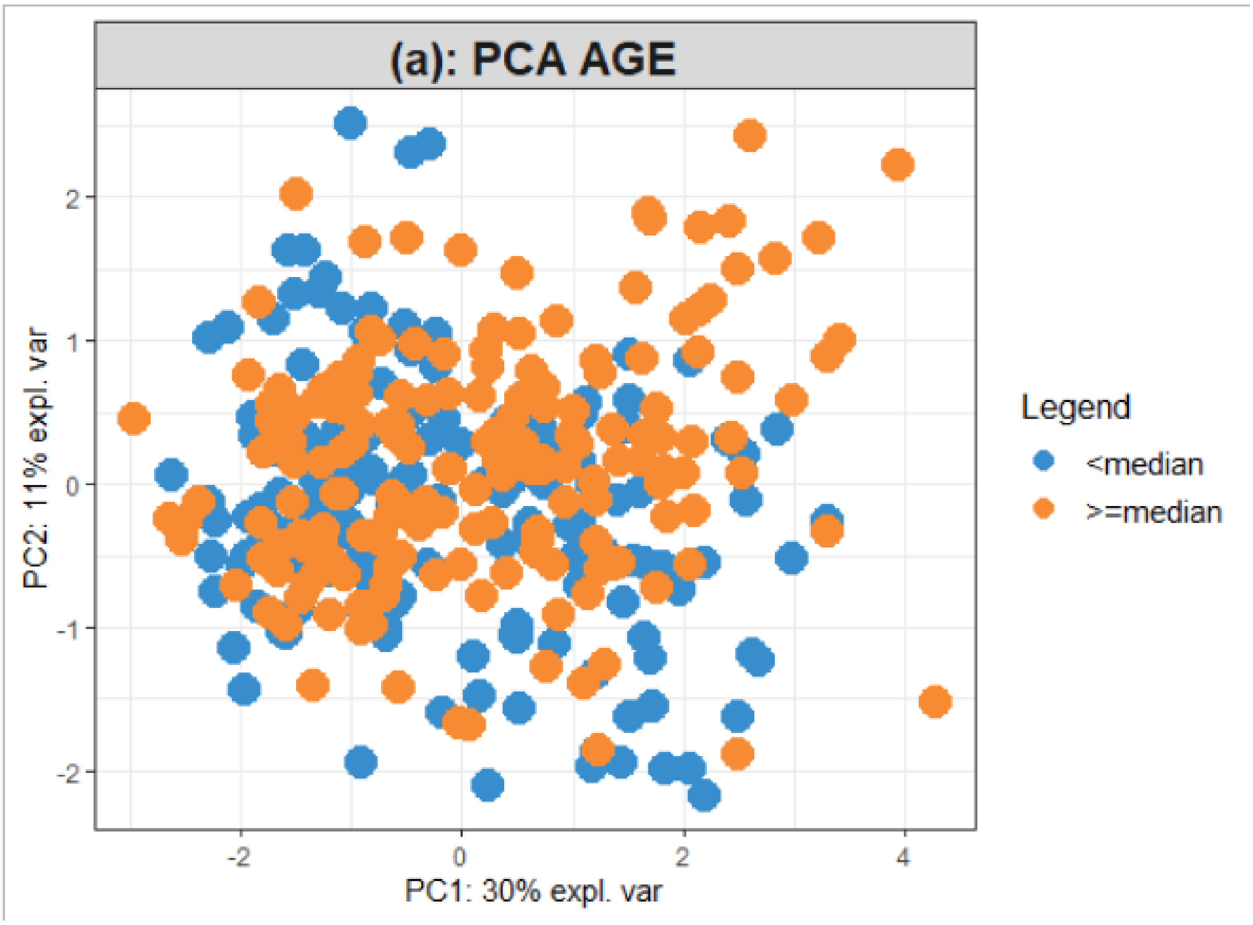

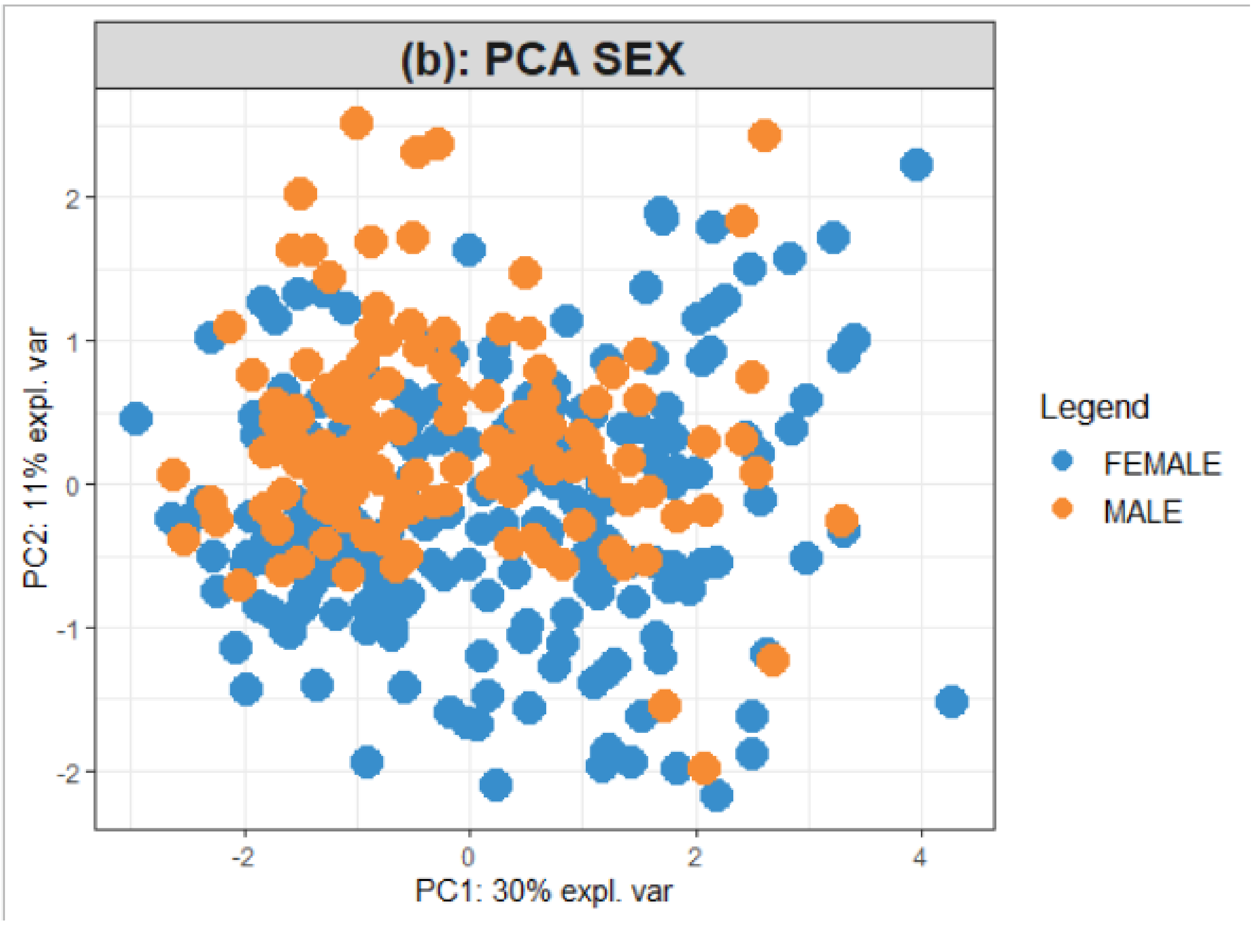
(b): PCA SEX
PC2: 11% expl. var
PC1: 30% expl. var
2
1
0
-1
-2
-2
0
2
4
Legend
FEMALE
MALE

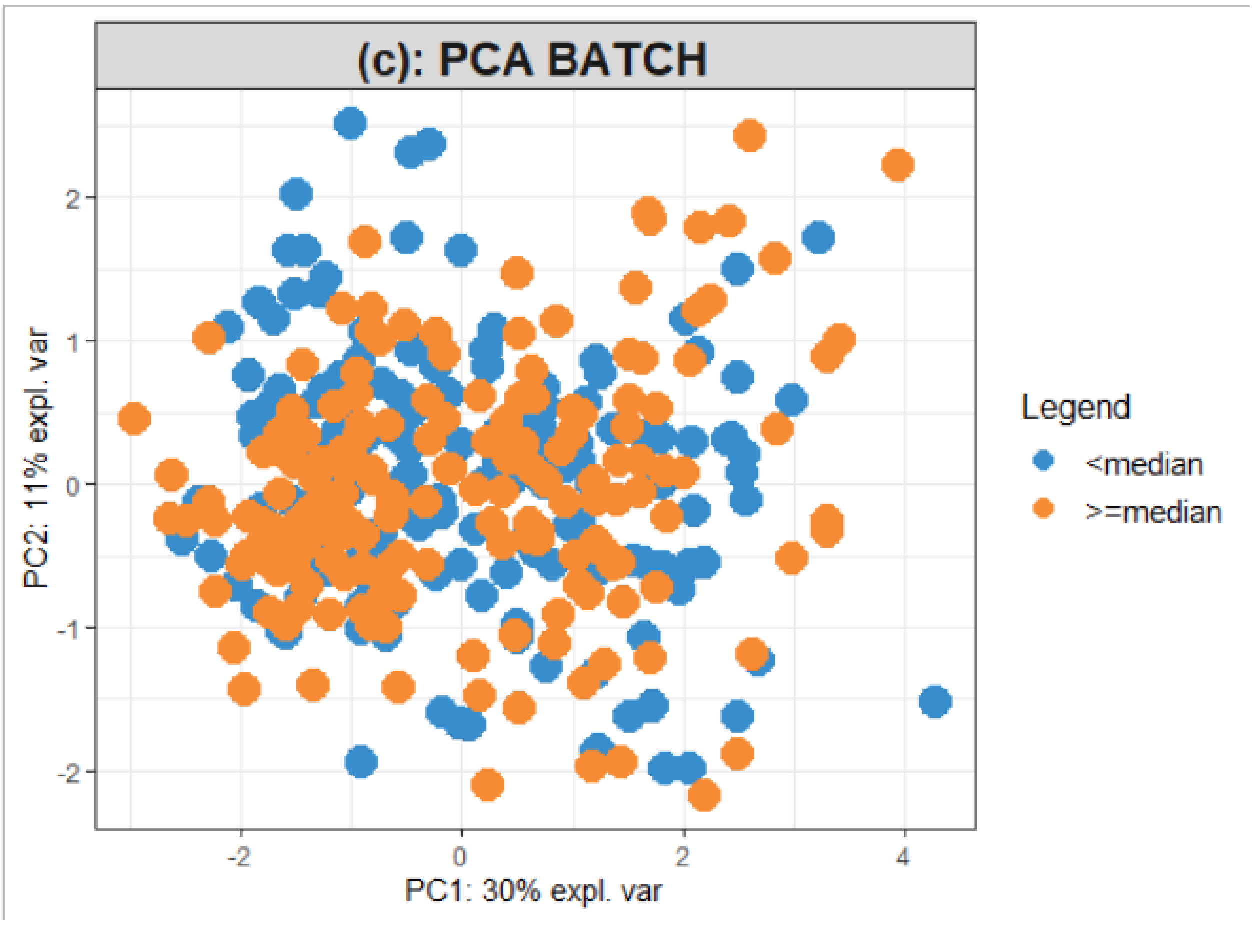
(c): PCA BATCH
PC2: 11% expl. var
PC1: 30% expl. var
2
1
0
-1
-2
-2
0
2
4
Legend
<median
>=median

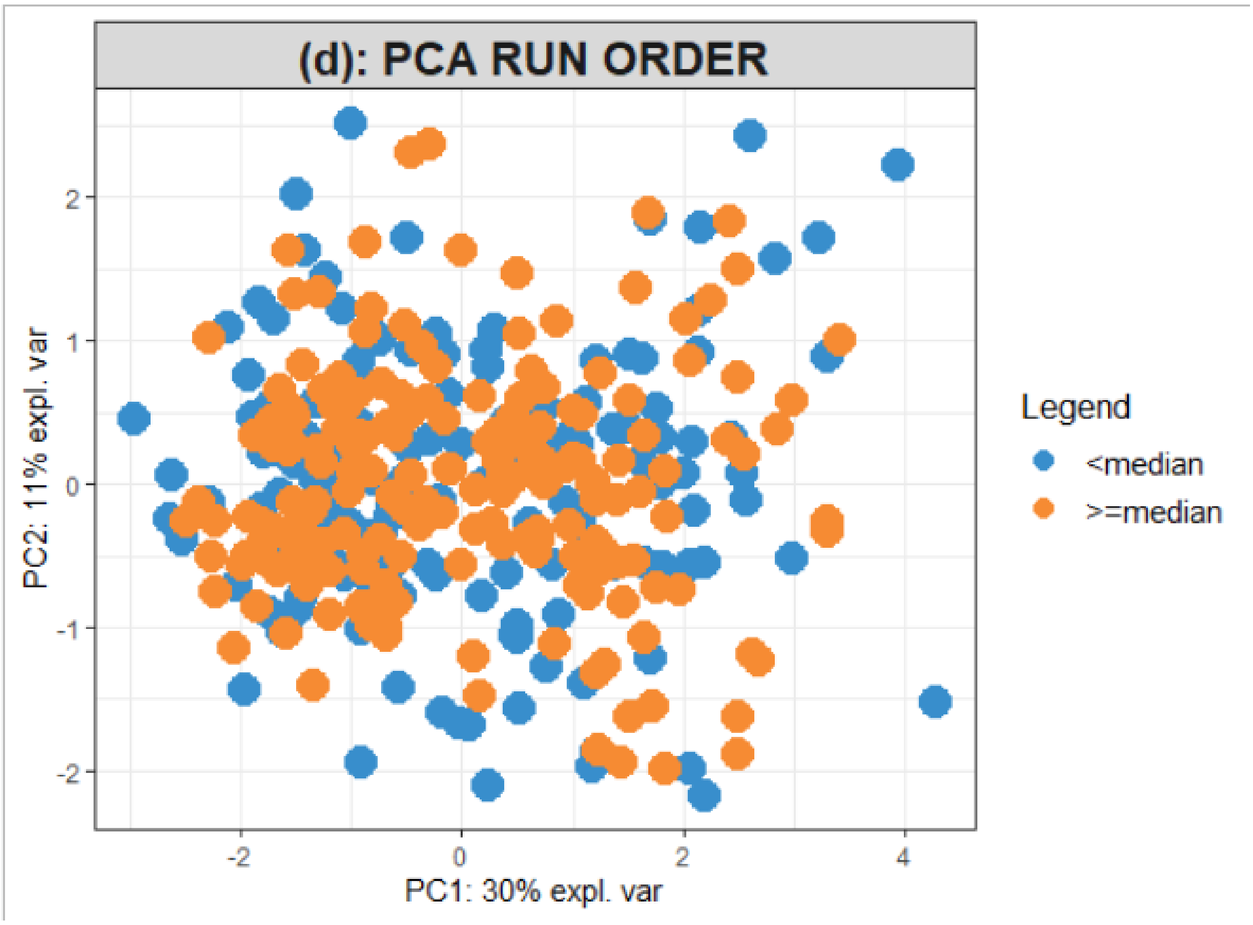
(d): PCA RUN ORDER
PC2: 11% expl. var
PC1: 30% expl. var
Legend
<median
>=median
2
1
0
-1
-2
-2
0
2
4

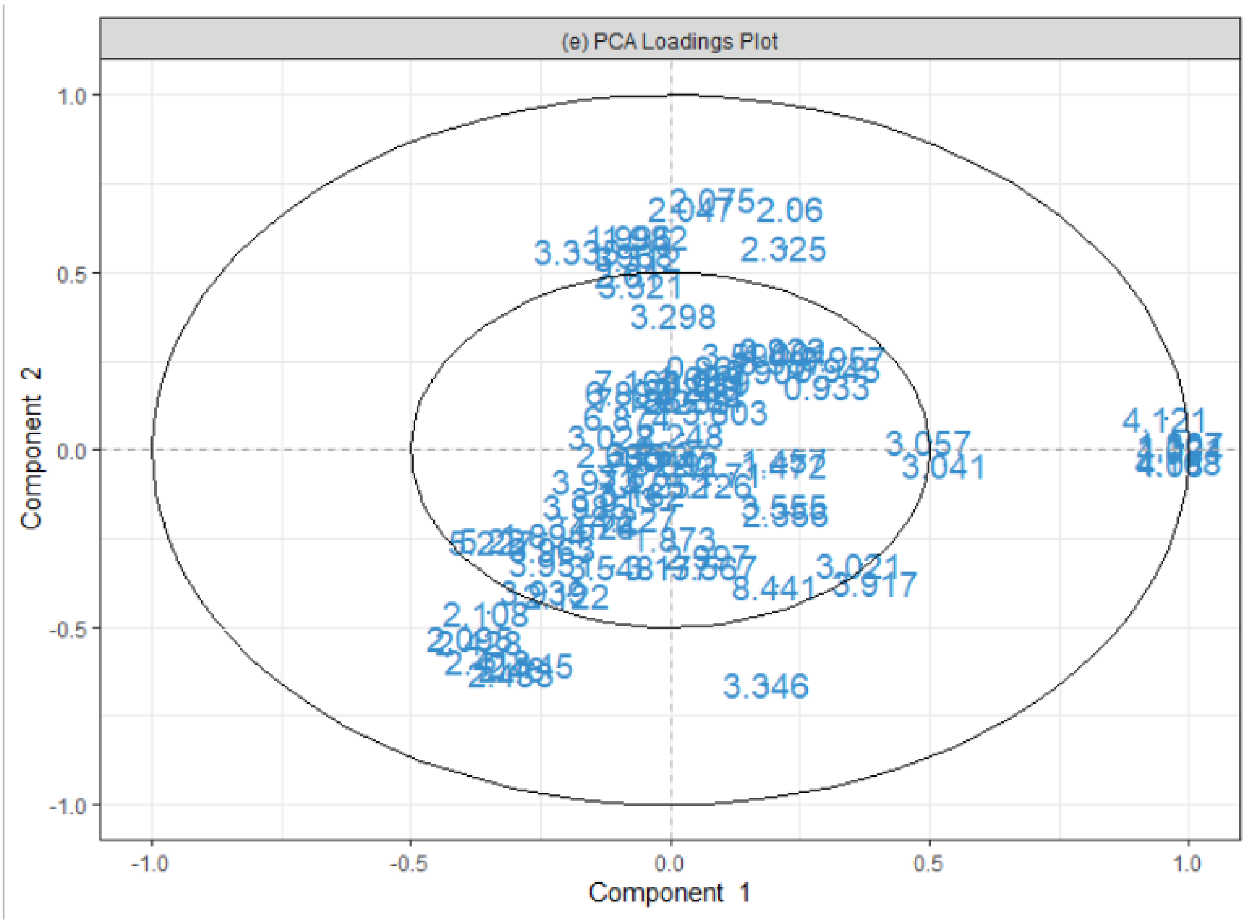

Figure S2: PCA score plots of the dataset that included all disease groups. Samples colored by (a) patient age, (b) patient sex, (c) analytical batch, (d) run order within batches. For continuous variables patient age, analytical batch and run order, groups were defined by the corresponding medians. Figure (e) is the loadings plot of the PCA score plot depicted in Figure 1. The clusters of samples can be explained by outstanding metabolites lactate (peaks at 1.3 and 4 ppm), methanol (3.346 ppm), glutamine (peaks at 2.1 and 2.4 ppm) and glutamate (peaks at 2.325 and 2.05 ppm).

Table S3: Summary of accuracies from analyses done via Approach A (ASCA correction).

Scenario	Analysis	Balanced Accuracy	Sensitivity*	Specificity**
EHT-PHT	sPLSDA	79[79-79]	84[83-84]	74[73-74]

	glmnet	77[77-78]	95[95-95]	60[60-60]
PA-PHT	sPLSDA	83[83-83]	89[89-90]	77[77-77]
	glmnet	83[83-84]	88[88-89]	78[78-79]
PPGL-PHT	sPLSDA	81[80-81]	86[85-87]	75[75-76]
	glmnet	80[80-81]	82[82-83]	78[77-78]

*Sensitivity is the true positive rate of the disease group (EHT, PA or PPGL)

**Specificity is the true positive rate of the control group (PHT).

All metrics are given as means, with the 95% confidence interval (in brackets).

Table S4: Summary of PLSDA analyses done to compare groups of samples defined by confounders.

PLSDA	B. ACCURACY	Group 1 TP Rate	Group 2 TP Rate
PA-PHT			
Cluster 1 vs. Cluster 2*	92[92-93]	89[89-90]	95[95-95]
Sample Age**	88[87-88]	90[89-90]	86[85-86]
PPGL-PHT			
Cluster 1 vs. Cluster 2*	93[93-93]	92[92-93]	94[93-94]
Sample Age**	80[80-80]	84[83-85]	76[75-77]

* Samples compared based on PCA cluster (Figure 1).

** Samples compared based on sample age, with the median value as the cutoff for the two groups.

All metrics are given as means, with the 95% confidence interval (in brackets).

Table S5: Summary of accuracies from analyses done via Approach B (after the exclusion of peaks related to confounders).

Scenario	Analysis	Balanced Accuracy	Sensitivity**	Specificity***	
EHT-PHT	sPLSDA	67[66-67]	69[69-70]	64[63-65]	
	glmnet	64[63-64]	87[86-87]	41[39-42]	
	Including patient age, sex, sPLSDA	68[67-68]	70[69-70]	66[65-66]	
	Including patient age, sex, glmnet	64[64-65]	88[87-88]	41[40-42]	
PA-PHT	sPLSDA	69[69-70]	70[69-71]	69[68-70]	
	glmnet	70[69-70]	71[70-71]	68[68-69]	
	Including patient age, sex, sPLSDA	69[69-70]	71[70-71]	68[67-69]	
	Including patient age, sex, glmnet	69[69-70]	70[69-71]	68[68-69]	
PPGL-PHT	sPLSDA	68[68-69]	69[68-70]	67[66-68]	
	glmnet	68[68-69]	65[64-65]	72[71-73]	

	Including patient age, sex, sPLSDA	69[69-70]	70[69-71]	69[68-70]		
	Including patient age, sex, glmnet	69[68-69]	65[64-65]	73[72-73]		
CS-PHT	sPLSDA	82[81-82]	79[78-80]	84[84-85]		
	glmnet	75[74-75]	53[51-54]	97[96-97]$		
	Including patient age, sex, sPLSDA	81[81-82]	78[76-79]	85[84-85]		
	Including patient age, sex, glmnet	74[73-75]	52[50-54]	95[95-96]		
		Balanced Accuracy	CS TP Rate	PA TP* Rate	PHT TP* Rate	PPGL TP* Rate
ALL-ALL	sPLSDA	53[52-53]	72[71-73]	53[52-54]	45[45-46]	42[41-43]
	glmnet	50[50-51]	39[38-40]	57[57-58]	59[58-60]	46[45-47]
	Including patient age, sex, sPLSDA	54[54-55]	79[78-80]	51[50-52]	45[44-46]	43[42-44]

	Including patient age, sex, glmnet	50[49-50]	41[39-42]	56[55-57]	58[57-58]	45[44-46]

*TP stands for True Positive

**Sensitivity is the TP rate of the disease group (EHT, PA, PPGL or CS)

***Specificity is the TP rate of the control group (PHT).

All metrics are given as means, with the 95% confidence interval (in brackets). The marked population of CS-PHT glmnet specificity estimate ($) was not normal (values close to 100).

Table S6: Summary of accuracies from analyses done via approach C (whole center exclusions).

Scenario	Analysis	Balanced Accuracy	Sensitivity**	Specificity***	
EHT-PHT	Cluster 1, sPLSDA	58[57-59]	62[61-63]	53[51-55]	
	Cluster 1, glmnet	50[50-50]	98[98-99]$	0[0-0]$	
	Cluster 1 + age, gender, sPLSDA	62[61-63]	64[63-64]	60[59-62]	
	Cluster 1 + age, gender, glmnet	50[50-50]	99[98-99]$	0[0-0]$	

PA-PHT	Cluster 1, sPLSDA	69[68-70]	77[76-79]	61[60-62]	
	Cluster 1, glmnet	68[68-69]	83[83-84]	53[52-55]	
	Cluster 1 + age, gender, sPLSDA	66[65-67]	68[66-69]	64[62-65]	
	Cluster 1 + age, gender, glmnet	65[64-66]	81[80-83]	48[46-50]	
PPGL-PHT	Cluster 1, sPLSDA	68[67-69]	69[68-70]	66[65-68]	
	Cluster 1, glmnet	70[69-70]	83[82-84]	56[54-57]	
	Cluster 1 + age, gender, sPLSDA	71[70-72]	73[72-74]	70[68-71]	
	Cluster 1 + age, gender, glmnet	69[68-70]	82[82-83]	55[54-57]	
		Balanced Accuracy	PA TP* Rate	PHT TP* Rate	PPGL TP* Rate

ALL-ALL	Cluster 1, sPLSDA	57[57-58]	69[67-70]	35[33-36]	69[68-70]
	Cluster 1, glmnet	58[57-59]	68[67-70]	30[29-32]	75[74-76]
	Cluster 1 + age, gender, sPLSDA	53[52-54]	54[53-56]	39[37-41]	66[65-67]
	Cluster 1 + age, gender, glmnet	56[56-57]	67[65-68]	27[25-29]	76[75-77]
PA-PPGL	Cluster 1 PAVPPGL, sPLSDA	79[78-80]	76[75-77]	-	81[80-83]
	Cluster 1 PAVPPGL, glmnet	77[76-78]	75[73-76]	-	80[78-81]
	Cluster 1 PAVPPGL + age, gender, sPLSDA	75[74-76]	73[72-74]	-	77[76-78]
	Cluster 1 PAVPPGL +	76[75-77]	73[72-74]	-	79[78-80]

	age, gender, glmnet				

*TP stands for True Positive

**Sensitivity is the TP rate of the disease group (EHT, PA, or PPGL)

***Specificity is the TP rate of the control group (PHT).

All metrics are given as means, with the 95% confidence interval (in brackets). The marked populations of model estimates ($) were not normal (values close to 100 or 0).

Table S7: Summary of accuracies from analyses done to evaluate datasets used in approach C (whole center exclusions).

Metric		EHT-PHT	PA-PHT	PPGL-PHT
	Balanced Accuracy	85[84-86]	84[83-84]	78[77-79]
	GYDR TP Rate*	80[79-81]	77[76-78]	80[79-81]
	ITTU3 TP Rate*	90[89-91]	90[89-91]	76[75-77]
+ age, sex	Balanced Accuracy	78[77-79]	79[78-80]	62[61-63]
	GYDR TP Rate*	74[73-75]	74[73-76]	71[70-72]
	ITTU3 TP Rate*	82[81-83]	84[83-84]	53[50-55]

*TP stands for True Positive

All metrics are given as means, with the 95% confidence interval (in brackets).

References

Sumner, L.W., Amberg, A., Barrett, D., Beale, M.H., Beger, R., Daykin, C.A., Fan, T.W.M., Fiehn, O., Goodacre, R., Griffin, J.L.,

Hankemeier, T., Hardy, N., Harnly, J., Higashi, R., Kopka, J., Lane, A.N., Lindon, J.C., Marriott, P., Nicholls, A.W., Reily, M.D., Thaden, J.J., Viant, M.R., 2007. Proposed minimum reporting standards for chemical analysis: Chemical Analysis Working Group (CAWG) Metabolomics Standards Initiative (MSI). Metabolomics 3, 211–221. https://doi.org/10.1007/s11306-007-0082-2
